# Editorial: Viral emerging and re-emerging diseases: basic understanding and future intervention strategies

**DOI:** 10.3389/fmicb.2024.1395742

**Published:** 2024-03-15

**Authors:** Mohamad S. Hakim, Wenshi Wang, Marco Goeijenbier, Shailendra K. Saxena

**Affiliations:** ^1^Department of Microbiology, Faculty of Medicine, Public Health, and Nursing, Universitas Gadjah Mada, Yogyakarta, Indonesia; ^2^Postgraduate School of Molecular Medicine, Erasmus MC-University Medical Center, Rotterdam, Netherlands; ^3^Viral Infection Working Group (VIWG), International Society of Antimicrobial Chemotherapy (ISAC), London, United Kingdom; ^4^Department of Pathogen Biology and Immunology, Jiangsu Key Laboratory of Immunity and Metabolism, Jiangsu International Laboratory of Immunity and Metabolism, Xuzhou Medical University, Xuzhou, China; ^5^Department of Intensive Care, Erasmus MC-University Medical Center, Rotterdam, Netherlands; ^6^Department of Intensive Care, Spaarne Gasthuis, Haarlem, Netherlands; ^7^Centre for Advanced Research, Faculty of Medicine, King George's Medical University, Lucknow, India; ^8^World Society for Virology, Northampton, MA, United States; ^9^The Indian Virological Society (IVS), New Delhi, India

**Keywords:** biology, control, emerging, vaccines, viral diseases

We have observed a recent pandemic of Coronavirus disease 2019 (COVID-19) due to an emerging virus, severe acute respiratory syndrome coronavirus 2 (SARS-CoV-2). SARS-CoV-2 is only one example of contagious virus transmitted to humans and significantly impacting on the global health and economy. In addition to SARS-CoV-2, many other viruses have recently emerged or re-emerged in the human population, including Middle East respiratory syndrome coronavirus (MERS-CoV) that is recently reported to continuously circulate in the kingdom of Saudi Arabia and the United Arab Emirates, Nipah virus (recently reported in India), Influenza A virus, Marburg virus (recently reported in Guinea and the United Republic of Tanzania), Measles, Dengue virus, among others. A close collaboration between the scientific community and public health authorities is highly required to better anticipate for the potential dangers of emerging and re-emerging viral diseases in the near future. A thorough understanding of the biology, immunology, and pathogenesis of viral infections is essential for the development of efficient prevention and treatment measures. Additionally, persistent genomic surveillance, identification of viral reservoirs, and vector management are critical.

The aim of the Research Topic was to present recent research on the growing subject of emerging and re-emerging viral diseases. In more detail, the Research Topic included comprehensive basic and translational aspects to better understand the genetics, diversity, immune pathogenesis, as well as the development of antiviral, vaccines, and diagnostic tools of emerging and re-emerging viral diseases. In addition to RNA viruses, we also focused on DNA viruses, such as monkeypox (mpox) virus and papillomaviruses. By highlighting these recent studies, we hope to improve our comprehension of the complexity of viral-host interactions and to more adequately prepare for upcoming large-scale viral outbreaks and pandemics.

Influenza viruses have been the cause of repeating epidemics and pandemics in the last century. Rafique et al. reviewed the global spreading of the H5N8 influenza virus. The highly pathogenic avian influenza (HPAI) subtype H5N8 was first reported in wild birds in China. To minimize the risk of HPAI incidence in the near future, strengthening biosecurity practice and increased active surveillance of wild birds are required. Additionally, to stop future HPAI H5N8 outbreaks, poultry birds in high-risk countries should receive vaccinations.

Recently, we experienced an epidemic due to monkeypox virus (MPXV). The virus could persistently survive on the environmental surfaces or in wastewater, enhancing its potential transmission. Thus, Taha et al. reviewed the persistence of MPXV on a variety of environmental surfaces. They also discussed various methods of disinfection and wastewater treatment to prevent transmission.

Detecting novel viruses or novel virus variants circulating in animals is important to identify potentially emerging viruses with epidemic or pandemic potential. Utilizing the advanced next generation sequencing (NGS) techniques, Li, Xiao et al. reported two novel strains of papillomaviruses, PV-HMU-1 and PV-HMU-2, in the nasal and throat swab samples collected from belugas *(Delphinapterus leucas)* at Polar Ocean Parks in Dalian and Qingdao. Li, Du et al. identified GI.1aP-GI.2 recombinants of rabbit hemorrhagic disease virus (RHDV) in domestic rabbits in China. RHDV is a highly infectious agent that causes acute multi-organ hemorrhagic syndrome with high morbidity and fatality rates in rabbits. Importantly, animal studies demonstrated that the recombinant GI.1aP-GI.2 variant had a moderately increased pathogenicity. Viral discovery in our Research Topic is also highlighted by the study of Xiao et al. that reported the first identification of Canine circovirus (CanineCV) in cats. They showed that about 9% of dogs and 3.4% of cats were CanineCV-positive. The whole genome analysis showed that the first cat-derived CanineCV belonged to the genotype 3. Xu et al. investigated the molecular epidemiology and virulence of goose astroviruses (GAstV). Phylogenetic studies revealed that the representative viruses belonged to genotype 2. Furthermore, subsequent *in vitro* and *in vivo* experiments showed notable variations in the virulence and pathogenicity across susceptible cells and embryos. While Zhang et al. examined viral-associated diarrhea from porcine diarrheal samples (including small intestinal contents and tissue, as well as feces) collected from five provinces in China from 2021 to 2023. They found that the most frequently detected virus was porcine epidemic diarrhea virus (PEDV), followed by porcine rotavirus (PRoV), porcine delta coronavirus (PDCoV), swine acute diarrhea syndrome coronavirus (SADS-CoV), and transmissible gastroenteritis coronavirus (transmissible gastroenteritis virus, TGEV). Interestingly, co-infections were commonly detected. All these findings emphasized the need of continuous surveillance in animals to identify novel pathogens or novel variants with altered pathogenic potential. Studies in human cases are also essential, as shown by Huang et al. that investigated the clinical picture of a novel bunyavirus infection causing severe fever with thrombocytopenia syndrome (SFTS) in infected patients.

Viral adaptation in humans following zoonotic transmission of emerging viruses is also an important aspect of virus evolution. By using the molecular dynamics simulation, Elli et al. examined the interactions between N1 neuraminidase (NA), particularly amino acid (aa) at position 347, with its substrates. Sialyllacto-N-tetraoses 3'SLN-LC and 6'SLN-LC are sialoglycan molecules correspond to the neuraminidase substrates in birds and humans, respectively. Their simulation demonstrated that tyrosine at position 347 played an essential role in the preference of neuraminidase binding for the avian-type substrates. They also emphasized the importance of an alterations at this position as a marker of host tropism (host-shift) and adaptive evolution of influenza viruses.

Our Research Topic also includes some studies on SARS-CoV-2. It is known that bats are the natural reservoir of coronaviruses (CoVs). Bat CoVs can occasionally jump to other mammals, including humans. Li, Tian et al. built a deep learning (DL) method to predict the evolutionary adaptation of bat CoVs to other mammals. The model can be utilized to predict transmission of bat CoVs to the human population. As the first step in viral infection, interaction between viral factors and host receptors is crucial before viral replication to ensue. Xia reviewed the identification of viral-host interactions by identifying host receptors for viral entry. He discussed the identification of ACE2 as the host receptor for SARS-CoV-2, as well as other candidate receptors and cofactors, including neuropilin-1 and integrin αVβ3. He then discussed the potential of the host receptors to be utilized as potential drug targets. In line, Zhou et al. established a novel hACE2 knock-in mouse model to mimic intestinal and respiratory SARS-CoV-2 infection. The generated mouse models were highly susceptible to intranasal SARS-CoV-2 infection. Interestingly, the infected mice not only developed lung diseases, but also acquired intestinal infection. Thus, this model is valuable in studying SARS-CoV-2 infection, particularly in the intestine.

Wang et al. tracked the first outbreak of SARS-CoV-2 Omicron BA.5.1.3 in Sanya city, the island province of Hainan, China. From August 1, 2022 to early September 2022, more than 8,500 cases have been confirmed as a result of the outbreak. Importantly, they found unique mutations, including three non-synonymous mutations in *ORF1ab* gene [Y1064N, S2844G, and R3574K within the non-structural proteins (nsp) 3, nsp4, and nsp6, respectively] and one synonymous mutation in *ORF3a* gene (S74S). The unique mutations identified in their study may result in more productive viral replication of the Omicron variant, but it deserves further exploration.

Negi et al. performed a retrospective study to search for prognosis indicators of SARS-CoV-2 infection. They examined the Ct values to evaluate viral load across three different waves of COVID-19 pandemic and correlated with demographic and clinical profiles of COVID-19 patients. During the second wave (mostly due to Delta variant, B.1.617), they observed a notably high percentage of patients with raised viral loads, indicating a higher severity. Their study suggested that in the event of infectious viral epidemic, the Ct value can be utilized as a monitoring tool for viral load and community transmission.

For antiviral development, Reshamwala et al. examined the antiviral properties of willow (*Salix* spp.) bark hot water extracts against enteroviruses and CoVs. Employing various molecular and cellular techniques, they revealed that the bark extracts of *Salix* spp. encompass a number of virucidal compounds that have the potential to exert antiviral actions both in direct and synergistic manner.

In conclusion, we need to improve our understanding for early detection and prevention of future threats of viral infectious diseases. We need to continuously identify viral reservoirs in the animal population, develop safe and efficacious vaccines against emerging and re-emerging viruses, as well as develop fast and efficient diagnostic tools and effective antiviral treatments.

## Author contributions

MH: Writing – original draft, Writing – review & editing. WW: Writing – review & editing. MG: Writing – review & editing. SS: Writing – original draft, Writing – review & editing.

